# The Design Features, Quality by Design Approach, Characterization, Therapeutic Applications, and Clinical Considerations of Transdermal Drug Delivery Systems—A Comprehensive Review

**DOI:** 10.3390/ph17101346

**Published:** 2024-10-09

**Authors:** Durgaramani Sivadasan, Osama A. Madkhali

**Affiliations:** Department of Pharmaceutics, College of Pharmacy, Jazan University, Jazan 45142, Saudi Arabia; dsivadasa@jazanu.edu.sa

**Keywords:** transdermal drug delivery system, skin permeation, permeation enhancers, quality by design approach, therapeutic applications

## Abstract

Transdermal drug delivery systems (TDDSs) are designed to administer a consistent and effective dose of an active pharmaceutical ingredient (API) through the patient’s skin. These pharmaceutical preparations are self-contained, discrete dosage forms designed to be placed topically on intact skin to release the active component at a controlled rate by penetrating the skin barriers. The API provides the continuous and prolonged administration of a substance at a consistent rate. TDDSs, or transdermal drug delivery systems, have gained significant attention as a non-invasive method of administering APIs to vulnerable patient populations, such as pediatric and geriatric patients. This approach is considered easy to administer and helps overcome the bioavailability issues associated with conventional drug delivery, which can be hindered by poor absorption and metabolism. A TDDS has various advantages compared to conventional methods of drug administration. It is less intrusive, more patient-friendly, and can circumvent first pass metabolism, as well as the corrosive acidic environment of the stomach, that happens when drugs are taken orally. Various approaches have been developed to enhance the transdermal permeability of different medicinal compounds. Recent improvements in TDDSs have enabled the accurate administration of APIs to their target sites by enhancing their penetration through the stratum corneum (SC), hence boosting the bioavailability of drugs throughout the body. Popular physical penetration augmentation methods covered in this review article include thermophoresis, iontophoresis, magnetophoresis, sonophoresis, needle-free injections, and microneedles. This review seeks to provide a concise overview of several methods employed in the production of TDDSs, as well as their evaluation, therapeutic uses, clinical considerations, and the current advancements intended to enhance the transdermal administration of drugs. These advancements have resulted in the development of intelligent, biodegradable, and highly efficient TDDSs.

## 1. Introduction

The transdermal drug delivery system (TDDS) has garnered significant attention due to its numerous advantages compared to conventional drug delivery systems. These advantages include its simplicity, predetermined dosing, ease of handling, and the ability for self-administration [1]. Transdermal delivery provides a competitive advantage over injectable and oral formulations by enhancing patient compliance and bypassing the first pass effect (metabolism) in the liver, improving bioavailability and efficacy. TDDS is a versatile pharmaceutical preparation mainly consisting of four layers (an impermeable backing membrane; a drug reservoir; a rate-limiting semipermeable membrane; and an adhesive layer), which can vary in size and contain one or more APIs. It is designed to be applied to the intact skin in order to be absorbed into the bloodstream [2]. The API is transdermally delivered into the bloodstream at a predetermined rate and then distributed throughout the body before reaching the intended location. This is known as transdermal administration, and drug delivery systems are known as transdermal therapeutic systems or transdermal drug delivery systems. Most of the TDDSs are designed to gradually release the active ingredients over many hours or days, once they are placed on the skin. The API initially permeates the SC and subsequently traverses the underlying epidermis and dermis without any drug buildup in the dermal layer [3]. Transdermal delivery offers both the controlled and continuous administration of pharmaceuticals, ensuring a steady supply of APIs. It is particularly beneficial for APIs with short biological half-lives, as it prevents the intermittent release of APIs into the bloodstream, which can lead to unwanted side effects. The quantifiable concentrations of the API in the blood, the detectable elimination of the drug and its byproducts in the urine, and the patient’s clinical reaction to the provided pharmacological treatment can be employed to ascertain the percutaneous drug absorption [4].

The restricted practical applicability of TDDSs is due to the low skin permeability of certain APIs. The layered physiological composition of the skin serves as the primary obstacle to transdermal drug delivery (TDD), and only certain active ingredients with particular physicochemical characteristics can permeate the skin and enter the systemic circulation [5]. The percutaneous absorption is influenced by various elements, including the drug’s physical and chemical qualities, such as molecular weight, solubility, partitioning coefficient, and dissociation constant. Additionally, the type of carrier vehicle, the temperature, and the circumstances of the skin also have a role. Subcutaneous blood vessels widen and the permeability coefficient increases with increasing temperatures; moist skin hydrates and relaxes the SC, improving drug absorption. Additionally, the SC’s blood flow, water content, and thickness vary according to race, gender, age, and location; these changes have an impact on the transdermal administration of drugs [6]. Various approaches and formulation strategies have been developed to enhance the transdermal permeability of APIs by overcoming the skin’s physiological barrier. In order to improve medication delivery, numerous nanocarriers such as exosomes, nanoparticles, nanolipids, nanoemulsions, and nanocrystals have recently been added to transdermal formulations. The transdermal preparations, when combined, exhibit encouraging safety and effectiveness.

The TDDS is composed of pressure-sensitive adhesives that ensure the adhesion of the preparation to the skin [7]. The product consists of a backing sheet that is impervious to both the active ingredient and water. Before placing the patch on the skin, it is necessary to remove the protective liner that covers the releasing surface of the patch. TDDSs have long been a subject of interest and have been utilized to administer APIs such as nicotine, fentanyl, nitroglycerin, and clonidine for the treatment of diverse ailments [8]. The constraints and disadvantages related to the bioavailability of conventional formulations justify the need for the development of TDDSs to improve drug delivery and penetration. The numerous advantages of various nanocarriers used in TDDSs have attracted significant interest from pharmaceutical experts and dermatologists [9]. Various APIs are offered in the form of TDDSs, and the specific location where these APIs are applied can differ according to their therapeutic classification [10]. The duration of drug release is also variable depending on the administration of these APIs. The advantages and limitations of TDDSs are shown in Table 1.

## 2. Skin Permeation as a Barrier

**Figure 1:**
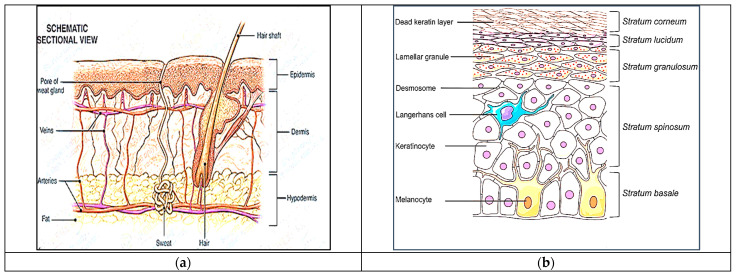
(**a**) Layers of the skin—a schematic sectional view. (**b**) The epidermis layer of the human skin. Adapted from [20], Springer, 2021.

It is essential to provide a concise overview of the function of human skin as a protective barrier to enhance the comprehension of the fundamental principles underlying the advancement of new technologies associated with TDDSs. The human skin, which makes up around 16% of an adult’s overall body weight, is the biggest organ in the body. Therefore, it has a crucial function in preserving homeostasis and serves as a barrier against external environmental dangers, chemically, physically, and biologically [15]. Human skin has a surface area of approximately 2 m^2^. It consists of five layers in the epidermis, namely the stratum corneum, stratum lucidum, stratum granulosum, stratum spinosum, and stratum basale. The outermost layer is formed by the SC, as shown in Figure 1a,b. The SC is a dense structure composed of fully matured keratinocytes that are spread out in a lipid-rich environment. It has a thickness of around 15–20 μm and is recognized as the step that controls the speed of absorption through the skin [16]. An undamaged and healthy SC serves as a barrier against both hydrophilic substances and big molecules. The SC covers the viable epidermis consisting of numerous skin layers made of viable keratinocytes, which is followed by the dermal layer consisting of connective tissues, fibroblasts, and other extracellular components such as hair follicles and glands. In order for the API included in the TDDS to enter the bloodstream, it must initially permeate through the layers of the skin. Several variables can influence the transdermal transfer of APIs, including skin permeability, surface area, application duration, and the metabolic activity of the skin [17]. Nonionic and moderately lipophilic APIs effectively accomplish sufficient absorption and penetration through the skin [17]. Transdermal drug delivery is restricted to active ingredients with a molecular weight of no more than 500 Daltons due to the SC’s resistance to molecular diffusion. Nano-drug delivery systems with advanced capabilities can effectively penetrate the epidermis and specifically target locations for treating skin-related illnesses [18]. These functional nanocarriers with a high degree of amphiphilicity effectively bypass the SC barrier by enhancing drug solubility and penetrating the skin, enabling the precise delivery of the desired dosage of the drug to the intended location [19].

### 2.1. Factors Affecting Transdermal Permeability

Transdermal drug delivery is not suitable for all APIs. The physical and chemical features of the drug, including its molecular weight, solubility, partition coefficient, and dissociation constant, as well as the type of the carrier vehicle and the condition of the skin, all contribute to percutaneous absorption. APIs within the molecular weight range of 400–500 Daltons, possessing sufficient lipid and water solubility, are capable of penetrating the skin [21]. The optimal molecular weight for an API intended for a TDDS should be approximately 400 Daltons or lower [22].

#### 2.1.1. Physicochemical Properties of APIs

- Solubility and partition coefficient

The solubility of an API directly affects its capacity to permeate the skin and impact the rate and degree of drug absorption. pKa is a measure of the drug’s ability to dissolve in a solvent, and the SC affects the movement of the drug from the solvent to the skin [23]. The log partition coefficient (logP) of the drug should be in the range of 1–3. Enhancing the lipophilic nature of the API can increase its ability to permeate the skin, allowing it to enter through the SC. However, it does not pass through the epidermis, since its water solubility has been reduced [24].

- pH condition and penetrant concentration

The acidic pH of the skin provides a defensive mechanism against microbes. The SC’s water-soluble components, perspiration and sebum secretions, and carbon dioxide elimination all affect the pH of the skin. Skin pH regulates the permeability barrier and maintains the cohesiveness and integrity of the SC. The penetration and absorption of unionized drugs can be affected by the pH of the skin. According to the pH partition hypothesis, only the unionized form of the drug can permeate through the lipid barrier in significant amounts. Hence, an optimal pH (4.1–5.8) level is beneficial and solutions with either a high or low pH might cause skin damage. The absorption rate increases as the concentration of penetrant in the vehicle increases optimally [25].

#### 2.1.2. Physicochemical Properties of Drug Delivery System

- Release characteristics and composition of drug delivery system

The release rate of a drug is determined by its lipid solubility in the vehicle. The rate of release and the permeability of APIs will be influenced through moisture and the interaction with skin lipids. The more the lipophilicity, the greater the drug absorption [26].

#### 2.1.3. Physiological and Pathological Conditions of Skin

- Lipid film and skin hydration

The lipid film functions as a barrier to inhibit the loss of moisture from the skin. The defeat of this coating will reduce the rate at which APIs are absorbed via the skin [27]. One way to obtain skin hydration is by applying plastic sheeting to the skin, which results in the buildup of sweat and condensed water vapors, improved porosity, and enhanced hydration [28].

- Effect of vehicle

The absorption of the API and its effect on the skin can be influenced by a vehicle through its impact on their physical state [29]. Paraffin-based products have a higher level of occlusiveness, whereas water-in-oil-based products have a lower level of occlusiveness. The presence of humectants in bases will cause skin dehydration and reduce the rate of absorption through the skin [30].

#### 2.1.4. Biological Factors

- Skin age and skin condition

The skin of a fetus, young individuals, and elderly individuals is more porous than the skin of adults. The viability of the epidermis is higher than that of the dermis. The local and systemic bioavailability of a topically administered API can be influenced if it undergoes metabolism during penetration [31].

- Species differences

The thickness of skin, appendage density, and keratinization of the skin vary from species to species, which affects the penetration.

- Regional skin site

The thickness of the skin, nature of the stratum corneum, and density of appendages vary from site to site. These factors affect the penetration significantly.

- Blood supply

Any changes in the peripheral circulation can also affect the transdermal absorption of the drugs.

## 3. Kinetics of Transdermal Permeation

**Figure 2:**
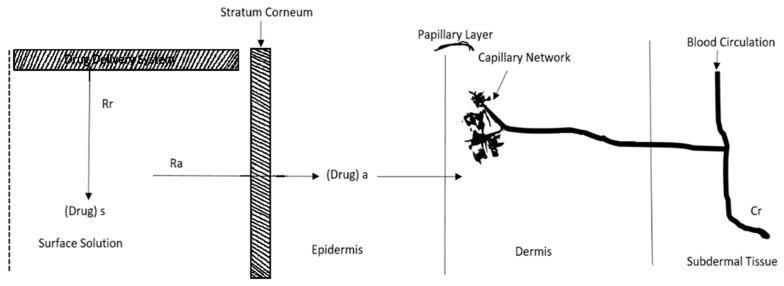
Schematic representation of the relationship between the rate of drug release (Rr) from a transdermal drug delivery system (TDDS) and the rate of drug absorption (Ra) by the skin. $C_{r}$ is the drug concentration in the body. Adapted from [35], CBS Publishers, 2005.

An understanding of the kinetics of skin permeation is crucial for the effective creation of transdermal therapeutic systems [32]. The process of transdermal API penetration consists of the following sequential steps, shown in Figure 2:

(a) Absorption by the outermost layer of the skin, known as the SC.
(b) Drug permeation through the viable outer layer of the skin.
(c) Absorption of the drug by the network of small blood vessels in the upper layer of the dermis [33].

Permeation is only possible if the API exhibits specific physicochemical features. The equation provided represents the rate at which a substance passes through the skin, known as the permeation rate (dQ/dt) [34]:

$$\frac{dQ}{dt}=P_{s}(C_{d}-C_{r})$$

where $C_{d}$ and $C_{r}$ are the concentrations of the skin penetrant in the donor compartment (on the surface of the SC) and in the receptor compartment (the body), respectively. $P_{s}$ is the overall permeability coefficient of the skin tissues. The permeability coefficient is given by the following relationship [34]:

$$P_{s}=\frac{K_{s}D_{ss}}{h_{s}}$$

$K_{s}$ represents the partition coefficient for the transfer of the penetrant molecule from a transdermal therapeutic system to the SC. $D_{ss}$ is the apparent diffusivity for the steady-state diffusion of the penetrant molecule through the skin tissues, and $h_{s}$ is the total thickness of the skin tissues. Given that $K_{s},\;D_{ss}$, and $h_{s}$ remain constant under the above conditions, it may be inferred that the permeability coefficient remains constant as well.

A constant rate of drug permeation can be obtained only when $C_{d}$ ≫ $C_{r}$; i.e., the concentration of the drug at the surface of the SC $\left(C_{d}\right)$ is consistently and substantially greater than the drug concentration in the body $\left(C_{r}\right)$. Then, the equation becomes the following [34]:

$$\frac{dQ}{dt}=P_{s}C_{d}$$

The rate of skin permeation (dQdt) is constant provided the magnitude of $C_{d}$ remains constant throughout the skin permeation [34]. Hence, the drug should be released from the device at a rate ($R_{r}$) that is either constant or greater than the rate of skin uptake ($R_{a}$); i.e., $R_{r}$ ≫ $R_{a}$, which is shown in Figure 2.

Since $R_{r}$ is greater than $R_{a}$, the drug concentration on the skin surface $C_{d}$ is maintained at a level equal to or greater than the equilibrium or saturation solubility of the drug in the SC $C_{s}$; i.e., $C_{d}$ ≫ $C_{s}$. Hence, a maximum rate of skin permeation [(dQdt)m] is obtained and is given by the following equation [34,36]:

dQdtm=PsCs

The equation above demonstrates that the highest rate of skin permeation is determined by the skin permeability coefficient ($P_{s}$) and its equilibrium solubility in the SC ($C_{s}$). Drug absorption via the skin seems to be restricted by the SC. The kinetics of skin permeation can be analyzed using in vitro investigations utilizing a freshly excised skin sample affixed to a precisely calibrated skin permeation cell [37].

## 4. Basic Components and the Classification of TDDSs

The TDDS consists of many components to facilitate the transport of the API from the skin to the bloodstream [38]. The backing layer functions as the outermost layer of the TDDS and protects the other layers from the surrounding environment. The backing layer must possess impermeability to both the API and penetration enhancers. The typical composition of this layer consists of a pliable and impermeable substance, such as polyethylene, polypropylene, or polyester [39,40]. The adhesive layer functions to affix the patch onto the skin and secure its position. The product is composed of a potent, pressure-responsive, hypoallergenic adhesive that is mild on the skin. Widely utilized adhesives include silicone adhesives and polyisobutylene- and polyacrylate-based adhesives [11]. The drug reservoir is the fundamental component of the TDDS. It comprises APIs that are administered transdermally. The formulation is designed to gradually release the API at a consistent pace throughout a specific duration. The drug release membrane regulates the rate of drug release from the patch [12]. Membranes consist of semipermeable substances that enable the regulated passage of APIs. Polyethylene sheets, an ethylene-vinyl acetate copolymer, and a cellulose acetate serve as the membranes that limit the rate of a process.

### 4.1. Polymer Matrices

The transdermal drug delivery system’s foundation is strengthened with the usage of polymers. Polymers are used to control the release rate of the drug and the adhesion or encapsulation of a drug reservoir in the TDDS. A polymer matrix can be prepared by the dispersion of the drug in liquid- or solid-state synthetic polymers. In order to fabricate a TDDS that effectively meets the various criteria, consideration must be given to the selection and design of the polymers. The polymer regulates the release of the API from the device. The polymer’s molecular weight, glass transition temperature, and chemical activity must be carefully selected to ensure the optimal diffusion and release of the specific API [41,42].

The ideal characteristics of the material include stability, non-reactivity with the drug, ease of manufacturing and fabrication, and cost-effectiveness. The polymer and its byproducts must be biocompatible with the host. The polymer’s mechanical characteristics should not undergo substantial deterioration when significant quantities of APIs are integrated into it [43].

Polyvinylpyrrolidone (PVP): N-vinyl pyrrolidone, the monomer, undergoes a polymerization reaction to yield polyvinyl pyrrolidone (PVP), a water-soluble polymer. PVP is biocompatible, biodegradable, inert, and soluble in water. PVP has been discovered to be appropriate for transdermal patches, because of its innate ability to create films. The usage of PVP is fraught with difficulties due to its intrinsic hydrophilicity and hygroscopicity. Because PVP films are hygroscopic, they absorb a lot of water vapor, which causes microbial contamination and renders the medicated patches dangerous or nearly useless. PVP and EC are combined to get around these problems and enhance the qualities and performance. When hydrophilic PVPs are combined with insoluble film formers like ethyl cellulose, the release rate constants of the former typically increase [44,45].

Ethylcellulose (EC): The water-insoluble polymer EC is utilized in controlled release dosage forms. EC is thought to be insoluble; however, it has the ability to absorb water. Its capacity to form hydrogen bonds with water is the reason behind this. In a porous, hydrophobic, polymeric drug delivery system, drug release happens when the drug dissolves in the bulk fluid that passes through the pores and diffuses through the pores that are filled with the media. The polymer chain dissolution process starts very slowly when the EC-based matrix patch comes into contact with an in vitro study fluid that is thermodynamically compatible with the polymer. The fluid is absorbed into the polymer matrix. It is commonly known that the release rate constant of an insoluble film former is increased upon the addition of a hydrophilic component. This could be the result of the dissolution of the aqueous soluble component of the film, which creates pores in the process and shortens the drug molecule’s mean diffusion path length [46].

Hydroxypropyl methylcellulose (HPMC): HPMC is a hydrophilic swellable polymer, which is also a matrix former in oral controlled release drug administration. Clear films have been produced using HPMC, presumably due to the drug’s sufficient solubility in the polymer. During dissolution tests, HPMC matrices without rate-controlling membranes showed a burst effect because the polymer was easily hydrated and swollen, causing the medication to release quickly [47].

Acrylic–acid matrices: Drug–polymer matrix films have been created using acrylic–acid matrices and plasticizers for transdermal delivery techniques. Eudragit RL PM, Eudragit S-100, Eudragit RS PM, and Eudragit E-100 are a few of the polymers that have been documented. A nonadhesive hydrophobic polymer called Eudragit NE-40D, a copolymer of methyl methacrylate and ethyl acrylate, has also been employed as a matrix former [48].

Cross-linked poly(ethylene glycol) (PEG): PEGs are the preferred polymers for many biological applications due to their biocompatibility. PEGs, cross-linked with tris(6-isocyanatohexyl) isocyanurate via a urethane–allophanate linkage, can transport proteins and create polymer networks that can swell in phosphate-buffered saline or ethanol and form a gel. It has been demonstrated that these systems release the solutes biphasically [49].

Organogels: Certain nonionic surfactants, including sorbitan monostearate, lecithin, and Tween, exhibit a propensity to form reverse micelles. Upon the addition of water, these surfactants in an organic solvent undergo associative reorientation to form a gel. These organogels can serve as a matrix for an enhanced transdermal medication delivery.

The polymers utilized in a TDDS, which encompass natural polymers, synthetic elastomers, and synthetic polymers, are shown in Table 2.

### 4.2. Drug Reservoir

The drug reservoir comprises drug particles that are either dissolved or dispersed throughout the matrix, facilitated by solvents and co-solvents. The selection of the drug for the effective development of the TDDS should be conducted with meticulous consideration [51]. Only potent APIs with a daily dosage in the range of a few milligrams per day (the dose should ideally be less than 10 mg per day) are appropriate. The half-life of the drug should be short (10 h or less), and the drug should not induce an allergic response. Drugs that undergo degradation in the gastrointestinal tract or are rendered inactive by the hepatic first pass effect are appropriate choices for TDDSs. Drugs that require prolonged administration or have adverse effects on tissues other than the intended target can also be developed as TDDSs.

### 4.3. Permeation Enhancers

Permeation enhancers are chemicals that modify the skin’s barrier to allow for the increased penetration of desired substances. The flux J of a drug across the skin can be written as follows [52]:

$$J=D\frac{dc}{dx}$$

The diffusion coefficient, denoted as D, is influenced by various factors such as the size, shape, and flexibility of the diffusing molecule, as well as the resistance of the membrane. The concentration of the diffusing molecules is represented by C, while x represents the spatial coordinate [53]. Permeation enhancers are believed to influence the stratum corneum in order to promote the penetration of substances into the skin. Several APIs have been examined for their capacity to increase the permeability of the SC [54]. Permeation enhancers are categorized in the following manner, as shown in Table 3.

### 4.4. Other Excipients Used in TDDSs [56]

Adhesives: The attachment of the TDDS to the skin has been achieved through the utilization of a pressure-sensitive adhesive. The adhesive is applied either on the front or rear of the device and extends around the edges. It must meet the following criteria: It should not cause irritation or sensitization to the skin upon contact. The API should firmly adhere to the skin throughout the dosing period without being displaced by activities. It should have a simple removal process and should not leave a permanent residue on the skin. It should come into direct contact with the skin. Commonly employed pressure-sensitive adhesives encompass polyisobutylenes, acrylics, and silicones.

Backing membrane: This is a material that is impervious and provides protection to the product when applied to the skin. Examples include a metallic plastic laminate, a plastic backing with absorbent pad, an occlusive base plate, and an adhesive foam pad (flexible polyurethane). They are flexible and provide a good bond to the drug reservoir, preventing the drug from leaving the dosage form through the top.

## 5. Approaches Used in the Development of TDDSs

**Figure 3:**
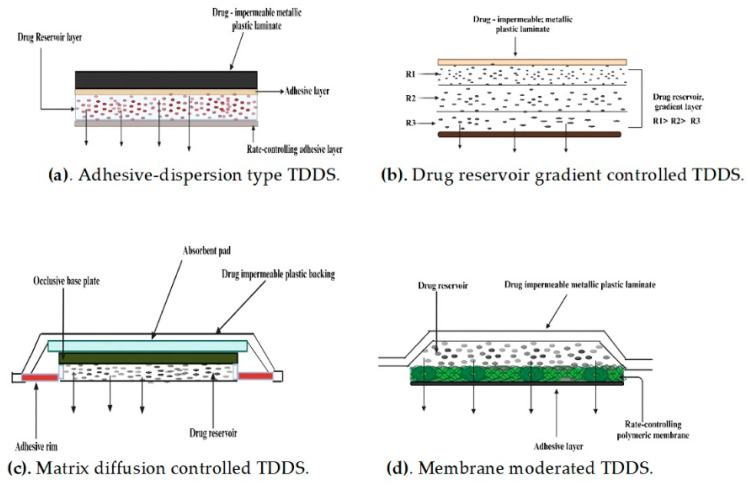
Approaches used in the development of TDDSs.

Transdermal dosage forms are composed of multiple layers, each with a distinct purpose, forming a fundamental structure. An impermeable covering keeps the system from becoming wet during use [57]. The second layer functions as a drug reservoir, providing a consistent supply of the API for a predetermined duration. Adjacent to the reservoir is the rate-controlling polymeric membrane that governs the rate at which an API is released over a predetermined period. The API administered by the system permeates the skin and reaches the systemic circulation. The drug delivery rate from the TDDS typically exceeds the maximum absorption capacity of the skin. Therefore, despite potential differences in skin permeability, a consistent rate of API absorption into the bloodstream is attained [58]. There exist four distinct approaches for acquiring a TDDS, as shown in Figure 3.

### 5.1. Membrane Permeation-Controlled Systems

The drug reservoir in this system is enclosed within a shallow compartment made of a drug-impermeable metallic plastic laminate. Additionally, there is a rate-controlling polymeric membrane that can be non-porous and has a certain drug permeability characteristic [59]. The APIs are exclusively allowed to be released via the rate-controlling membrane. The drug particles in the drug reservoir compartment are either disseminated within a solid polymer matrix or suspended in a non-leachable, viscous liquid medium, such as silicone fluid, resulting in the formation of a paste-like suspension [60]. To ensure close contact between the TDDS and the skin, a thin coating of an adhesive polymer that is compatible with the API and hypoallergenic, such as silicone or polyacrylate adhesive, can be placed on the outer surface of the membrane that controls the rate of drug release. The drug release rate of this particular TDDS can be adjusted by altering the polymer composition, permeability coefficient, thickness of the rate-limiting membrane, and adhesives [61]. The continuous dispersion of APIs is the primary benefit of this approach. However, there exists the potential for an inadvertent rupture of the rate-controlling membrane, resulting in dose dumping or the abrupt release of the complete drug content [62]. A few examples of this system are shown in Table 4.

The intrinsic rate of drug release from this type of drug delivery system is explained by the following equation [20]:

dQdt=CR1Pm+1Pa

where C_R_ is the concentration of the drug in the reservoir compartment. P_a_ and P_m_ are the permeability coefficients of the adhesive layer and the rate-controlling membrane, respectively. P_a_ and P_m_ are defined as follows [20]:

$$P_{m}=\frac{K_{m/r.}D_{m}}{h_{m}}$$

$$P_{a}=\frac{K_{a/m.}D_{a}}{h_{a}}$$

$K_{m/r}$ and $K_{a/m}$ are the partition coefficients for the interfacial partitioning of the drug from the reservoir to the membrane and from the membrane to the adhesive, respectively; $D_{m}$ and $D_{a}$ are the diffusion coefficients in the rate-controlling membrane and adhesive layer, respectively; and $h_{m}$ and $h_{a}$ are the thickness of the rate-controlling membrane and adhesive layer, respectively. Substituting the equations for $P_{a}$ and $P_{m}$, the equation for the intrinsic rate of drug release ($\frac{dQ}{dt}$) becomes the following [20]:

$$\frac{dQ}{dt}=\frac{K_{m/r.}K_{a/m.}D_{m}.D_{a}}{K_{m/r.}D_{m}.h_{a}+K_{a/m.}D_{a}.h_{m}}C_{R}$$

The above equation defines the intrinsic rate of drug release from a membrane-moderated drug delivery system.

### 5.2. Adhesive Dispersion-Type Systems

In this particular TDDS, the drug reservoir is produced by directly dispersing the drug in an adhesive polymer, such as a poly(isobutylene) or poly(acrylate) adhesive [67]. The medicated adhesive is then spread onto the flat sheet of a drug-impermeable metallic plastic backing using either solvent casting or hot melt techniques, forming a thin drug reservoir layer. Above the layer containing the APIs, thin layers of a non-medicated, adhesive polymer with a specified permeability and consistent thickness are applied to develop an adhesive diffusion-controlled drug delivery system.

An example of this type of system is the isosorbide dinitrate-releasing TDDS for the once-a-day medication of angina pectoris and the transdermal controlled administration of verapamil. The rate of drug release in this system is defined by the following [20]:

$$\frac{dQ}{dt}=\frac{K_{a/r.}D_{a}}{h_{a}}C_{R}$$

$K_{a/r}$ is the partition coefficient for the interfacial partitioning of the drug from the reservoir layer to the adhesive layer [68].

This particular TDDS can also be altered to allow for the manipulation of the drug loading level in small increments, resulting in the formation of a gradient of the drug reservoir across the multilaminate adhesive layers. An example of this system is the nitroglycerin-releasing TDDS.

The drug release rate in this system is defined by the following [20]:

$$\frac{dQ}{dt}=\frac{K_{a/r.}D_{a}}{h_{a}\left(t\right)}A\left(h_{a}\right)$$

$h_{a}\left(t\right)$ is the thickness of the adhesive layer for the diffusion of the drug molecules with time. To compensate for this time-dependent increase in the diffusional path due to the depletion of the drug dose by release, the drug loading level is also increased with the thickness of the diffusional path $A\left(h_{a}\right)$ to achieve a constant drug release profile [69,70].

### 5.3. Matrix Diffusion-Controlled Systems

The drug reservoir in this system is produced by evenly distributing the drug particles within a polymer matrix that is either hydrophilic or lipophilic. Subsequently, the medicated polymer is molded into a medicated disc, possessing a specific surface area and controlled thickness [71]. The drug particles can be dispersed in the polymer matrix through two methods: either by uniformly mixing finely ground drug particles with a liquid polymer or a highly viscous base polymer and then cross-linking the polymer chains or by homogeneously blending the drug solids with a rubbery polymer at a high temperature. The drug reservoir can also be created by dissolving the drug and polymer in a common solvent and then evaporating the solvent in a mold at a higher temperature or in a vacuum [72]. The polymer disc, which holds the drug, is then attached to an occlusive base plate within a compartment made of a drug-impermeable plastic backing. The adhesive polymer is thereafter applied evenly around the circumference to create a band of the adhesive rim around the medicated disc. A good example of this technology is the transdermal therapeutic system that releases nitroglycerin and delivers it continuously through the skin [73].

The rate of drug release from this system is defined as follows [20]:

$$\frac{dQ}{dt}={\left[\frac{AC_{p}D_{p}}{2t}\right]}^{1/2}$$

where *A* is the initial loading dose dispersed in the polymer matrix. $C_{p}\;\mathrm{and}\;D_{p}$ are the solubility and diffusivity of the drug in the polymer, respectively.

### 5.4. Microreservoir-Type or Microsealed Dissolution-Controlled Systems

This system is a hybrid of the reservoir and matrix diffusion drug delivery methods. The drug reservoir is produced by combining the drug solids with a water-soluble liquid polymer solution, and then uniformly dispersing the drug suspension within a lipophilic polymer using a high-energy dispersion technique [74]. This process results in the formation of distinct and non-leachable microspheres that serve as the drug reservoir. The rapid stabilization of this thermodynamically unstable dispersion is achieved by promptly cross-linking the polymer chains in situ, resulting in the formation of a medicated polymer disc with a consistent surface area and fixed thickness. If necessary, the device might be additionally coated with a biocompatible polymer layer to alter the method and rate of drug release [30]. A transdermal therapeutic system is created by placing the medicated disc in the middle and surrounding it with an adhesive rim. A specific example of this kind of technology is the TDDS that releases nitroglycerin for the treatment of angina pectoris once a day. The microreservoir technology exhibits zero-order release kinetics while mitigating the risk of dose dumping [31].

The rate of release of drugs from the microreservoir system is defined by the following [20]:

$$\frac{dQ}{dt}=\frac{D_{p}D_{d}.m.K_{p}}{D_{p}h_{d}+D_{d}h_{p}.m.K_{p}}\left[n.S_{p}\frac{D_{l}S_{l}.\left(1-n\right)}{h_{l}}\left(\frac{1}{K_{l}}+\frac{1}{K_{m}}\right)\right]$$

where m = a/b, with a representing the ratio of the drug concentration in the bulk of the elution medium and b representing the drug concentration at the outer edge of the polymer coating [75]. n represents the quotient of the drug concentration at the inner boundary of the interfacial barrier divided by the drug solubility in the polymer matrix. The drug diffusivities in the liquid layer surrounding the drug particles, the polymer coating membrane covering the polymer matrix, and the hydrodynamic diffusion layer surrounding the polymer coating are represented by $D_{l},D_{p\;},\;\mathrm{and}\;D_{d}$, respectively. The thickness of these layers is denoted by $h_{p}$, $h_{l},\;\mathrm{and}\;h_{d}$. $K_{l},K_{m},\;\mathrm{and}\;K_{p}$ are the partition coefficients that describe the distribution of the drug between different compartments, specifically from the liquid compartment to the polymer matrix, from the polymer matrix to the polymer coating membrane, and from the polymer coating membrane to the elution solution, respectively. $S_{l}\;\mathrm{and}\;S_{p}$ represent the drug’s solubilities in the liquid compartment and the polymer matrix, respectively. This drug delivery system follows either a partition control or matrix diffusion control mechanism [76].

## 6. Production of TDDSs

The production of TDDSs on a large scale requires the use of manufacturing technologies that are either novel or adapted from the conventional pharmaceutical sector.

### 6.1. Asymmetric TPX Membrane Method

The backing membrane consists of a heat-sealable polyester film, specifically type 1009, with a diameter of 1 cm and a concave shape. The drug sample is placed into the concave membrane, which is then coated with a TPX poly (4-methyl-1-pentene) asymmetric membrane and sealed with an adhesive layer [77]. To produce a TPX asymmetric membrane, either a wet inversion method or a dry inversion approach is employed.

### 6.2. Circular Teflon Mold Method

Polymers are dissolved in an organic solvent in different proportions to create solutions. The determined quantity of the drug is dissolved in half the volume of the identical organic solvent. The permeation enhancers are dissolved in the remaining portion of the organic solvent and subsequently combined [78]. Di-N-butyl phthalate serves as a plasticizer and is included in a drug–polymer solution. The entire mixture should be agitated for a duration of 12 h and thereafter transferred into a round Teflon mold. The molds should be positioned on a flat surface and covered with an inverted funnel to regulate the evaporation of the solvent in a laminar flow hood model with an air velocity of 0.5 m/s. The solvent is allowed to evaporate for a duration of 24 h. The films that have been stored need to be kept for a further 24 h at a temperature of 25 ± 0.5° in a desiccator that contains silica gel. The films must be assessed within a week of their preparation [79].

### 6.3. Mercury Substrate Method

The medicament is dissolved or dispersed in a solution of a polymer comprising plasticizer. The solution is then agitated for 10–15 min to ensure a consistent, homogeneous mixture and poured into a flat surface of mercury with a covering of an inverted funnel to avoid evaporation of the solvent [80]. The consistent release of the medication via the skin is dependent on the formation of a homogenous film, which is facilitated by this procedure.

### 6.4. By Using the IPM Membrane Method

This technique involves dispersing the drug in a solution composed of water and propylene glycol, which also contains carbomer 940 polymers. The mixture is then agitated for a duration of 12 h using a magnetic stirrer. To counteract the dispersion, triethanolamine is added to neutralize it and increase its viscosity. A solution gel can be generated by using a buffer with a pH of 7.4, particularly when the drug’s solubility in an aqueous solution is extremely low. The gel that is produced is incorporated into the IPM membrane [81].

### 6.5. By Using the Ethylene Vinyl Acetate Copolymer (EVAC) Membrane Method

To develop the desired transdermal therapeutic system using this method, one can utilize a 1% Carbopol reservoir gel along with polyethylene (PE) and EVAC membranes as the rate-controlling membranes. When the API is not able to dissolve in water, propylene glycol is employed to create the gel formulation. The API is solubilized in propylene glycol. A Carbopol resin is introduced into the aforementioned solution and neutralized using a 5% *w*/*w* solution of sodium hydroxide. The gel-based drug is applied onto a backing layer sheet that covers the designated area. A rate-limiting membrane is applied onto the gel and the edges are sealed using heat to achieve a device that is impermeable to leaks [82].

### 6.6. Aluminum-Backed Adhesive Film Method

The aluminum-backed adhesive film approach is appropriate when the initial dose of the API exceeds 10 mg. Chloroform is employed as a solvent in this procedure due to its ability to dissolve a wide range of APIs and adhesives. The drug is dissolved in chloroform, and an adhesive material is added to the drug solution and dissolved. An individually crafted aluminum mold is coated with aluminum foil and sealed at the ends with snugly fitting cork blocks [83].

### 6.7. By Using the Free Film Method

The production of cellulose acetate-free film involves the application of chloroform over a mercury surface. A polymer solution with a concentration of 2% *w*/*w* is prepared. A plasticizer is incorporated into the polymer at a concentration of 40% by weight. A glass ring is positioned on a glass Petri dish, and then mercury is poured onto the surface. Subsequently, 5 mL of the polymer solution is dispensed onto the glass ring. A funnel is positioned on a Petri plate to regulate the rate of solvent evaporation. A thin layer develops on the surface of the mercury once the solvent has fully evaporated. The dehydrated coating is thereafter divided and preserved in a desiccator, sandwiched between sheets of wax paper until it is required [83].

## 7. Enhancement of the Transdermal Drug Delivery

**Figure 4:**
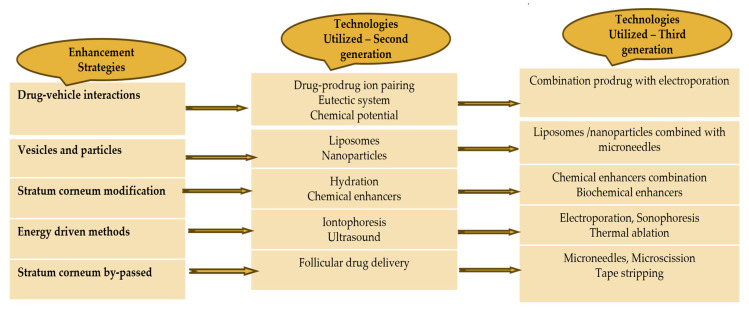
Enhancement methods of TDDSs.

In order to improve absorption via the skin and increase the effectiveness of the therapy, APIs need to possess a low molecular weight (less than 1 kDa), an affinity towards both lipid-based and water-based substances, a short duration of action, and a non-irritating effect on the skin. Several variables influence the ability of APIs to pass through the skin, including variations across species, the length of exposure, the age and site of the skin, the temperature of the skin, moisture levels in the skin, pre-treatment techniques, the application region, the condition of the skin, and the physical properties of the substance being absorbed [84]. Various enhancement methods are shown in Figure 4.

### 7.1. Active Drug Delivery (Using Equipment)

External stimuli, such as electrical, mechanical, or physical stimulation, have been found to increase the ability of APIs and biomolecules to pass through the skin, compared to applying APIs topically. Active transdermal delivery, also known as TDDS with appropriate equipment, efficiently and consistently delivers APIs into the skin at a rapid pace. This kind of improved TDDS can expedite the therapeutic effectiveness of administered APIs [85]. The various delivery methods include iontophoresis, sonophoresis, photochemical waves, electroporation, microneedles, and thermal ablation.

#### 7.1.1. Iontophoresis [86]

Iontophoresis is the process of employing an electrical field (less than 0.5 mA/cm^2^), to transfer a charged chemical substance across the skin’s membrane. As a way to administer peptides and proteins, an iontophoresis-enhanced TDDS has demonstrated considerable potential. By applying an electrochemical potential gradient, this method has been used to transport ionic or nonionic drugs in vivo. The effectiveness of iontophoresis depends on the polarity, valency, and mobility of the therapeutic molecules, the type of applied electrical cycle, and the drug formulation.

#### 7.1.2. Sonophoresis [86]

The transdermal administration of drugs also employs low-frequency sonophoresis, which involves the application of low-frequency (20–100 kHz) ultrasonic waves as an active way of stimulation. High-frequency ultrasound is another technique being researched to enhance TDDSs, which has the potential to alter the SC’s integrity and penetrability. This technique has been used to administer a variety of pharmaceuticals of different classes, including high-molecular weight medications like insulin and mannitol, regardless of their solubility, dissociation and ionization constants, and electrical properties (including hydrophilicity).

#### 7.1.3. Electroporation

Another new technique that uses tiny electrical impulses to administer drugs is called electroporation. This enhances the penetration of hydrophilic drugs to the SC. This technique creates tiny pores in the SC that increase permeability and facilitate drug diffusion by applying high-voltage electric pulses to the skin for brief periods of time (between 5 and 500 V). This skin permeabilization treatment is extremely safe and painless. The drawbacks of this approach include tiny delivery loads; severe cellular disruption, which can occasionally result in cell death; drug degradation from heating; and protein denaturation [87].

#### 7.1.4. Thermal Ablation [88]

Thermal ablation can be induced by laser and radiofrequency methods depending on the different sources of thermal energy [89]. Thermal ablation is typically facilitated by laser and radiofrequency techniques, contingent upon the various sources of thermal energy. Laser thermal ablation techniques employ a laser to create micropore structures in the skin and elevate the skin temperature, hence enhancing skin diffusivity. Radiofrequency thermal ablation entails the insertion of a series of needle-like metallic microelectrodes directly into the skin, followed by the application of a high-frequency electric current within the radiofrequency range of 100–500 kHz, resulting in the formation of micron-scale channels in the stratum corneum. The ablation of the stratum corneum via thermal means necessitates temperatures over 100 °C, resulting in the heating and evaporation of keratin. The extent of the modification of the stratum corneum structure correlates with the locally increased temperature, suggesting that it is an optimal method for precise drug delivery management.

#### 7.1.5. Microneedles

The microneedle drug delivery system is an innovative method for administering pharmaceuticals directly into the circulatory system via a needle. This entails a method wherein micron-sized needles penetrate the superficial layer of the skin, facilitating drug diffusion through the epidermis. Due to their brevity and slenderness, these microneedles administer medications directly to the blood capillary region for effective absorption, hence mitigating discomfort. The development of microneedle systems has been extensively studied, taking into account the objectives, drug types, dosages, and intended targets for application. To date, microneedles can be produced using laser-mediated methods and photolithography. Three-dimensional printing, micro-stereolithography, and two-photon polymerization have also been examined for the fabrication of diverse microneedle systems. This approach is highly favored for transdermal medication delivery and remains a prominent focus of contemporary research [90].

#### 7.1.6. Photomechanical Waves

Photodynamic waves directed at the skin can penetrate the stratum corneum, facilitating the passage of the medicine through the temporarily formed channel. The incident wave results in little ablation, accomplished through low radiation exposure of roughly 5–7 J/cm^2^ to enhance the depth to 50–400 μm for effective transmission. This restricted ablation demonstrates a prolonged rise and duration relative to previous direct ablation methods, necessitating the regulation of photodynamic wave characteristics to ensure the product’s delivery to the desired skin depth [91].

### 7.2. Passive Drug Delivery (Using Chemical Enhancers)

Chemical penetration enhancers (CPEs) facilitate the diffusion of APIs through the skin or enhance their ability to dissolve in the skin, resulting in the development of highly potent formulations, microemulsions, nanoemulsions, polymeric nanoparticles, and vesicles. Their action is accomplished by disrupting the structured lipid bilayer, interacting with the proteins in the cell membrane and intercellular proteins, disrupting the lipids between cells, increasing moisture in the outermost layer of the skin, and improving the partition coefficient of the APIs. Penetration enhancers can be utilized either independently or in conjunction with chemical penetration enhancers to provide enhanced skin penetration, surpassing the effectiveness of individual chemicals. Over 300 chemical penetration enhancers have been employed in various TDDSs to aid the transportation of pharmaceuticals over the SC. An ideal enhancer should possess the qualities of being non-toxic and biocompatible, while also exhibiting predictable and consistent activity and duration of effect [91]. Vesicles: These are made up of amphiphilic molecules arranged in a bilayer configuration; vesicles are colloidal particles packed with water. To achieve transdermal absorption, they can transport APIs that are soluble in fat or water. Vesicle-like liposomes, transferosomes, and ethosomes can be used in TDDSs to regulate the rate of absorption by creating a multilayered structure. Because of their unique shape, liposomes may encapsulate both fat-soluble and water-soluble drugs and are both hydrophilic and hydrophobic. Nevertheless, some research has demonstrated that liposomes are limited to the skin’s surface and are unable to penetrate the epidermis’s granular layer, which reduces the quantity of API that is absorbed into the bloodstream. This characteristic makes APIs more likely to remain on the skin, to remain active for longer at the site of the lesion, and to provide sustained release over an extended period of time. As a result, the system of choice for the topical therapy of skin conditions is liposomes [92].

#### 7.2.1. Nanoemulsions [93]

Nanoemulsions are frequently utilized for the transdermal delivery of hydrophilic and hydrophobic drugs that have problems with lipophilicity, bioavailability, and solubility. They are characterized by a low viscosity and by being isotropic, kinetically stable, and thermodynamically metastable with a mean droplet size that ranges from approximately 1 to 100 nm. They consist of transparent or translucent oil globules mixed with an aqueous phase and stabilized by an interfacial membrane made of very small droplet-sized surfactant or co-surfactant molecules. Excellent wettability is ensured by the nano-size droplets, large specific surface area, and low surface tension of nanoemulsions, which also guarantee close contact with the skin. Numerous other advantages of nanoemulsions include their extended shelf life, high solubilization capacity, drug stability until they reach the desired site of action, enhanced bioavailability, ease of preparation, and low energy input during production. Similar components, though in varying ratios, are shared by nanoemulsions and microemulsions. However, their main difference is their droplet shape, size distribution, and kinetic stability. Compared to topical skin preparations, nanoemulsions show a shorter transdermal time and improved transdermal absorption.

#### 7.2.2. Polymeric Nanoparticles [94]

Nanoparticles (NPs) are nanocarriers that range in size from 1 to 1000 nm. When drugs are administered as nanoparticles (NPs), their release behavior is targeted and controlled, the drug’s in vivo dynamics are altered, and the drug’s blood residence duration is prolonged. All of these effects improve the drug’s bioavailability and decrease its toxicity and adverse effects. Polymeric NPs are attracting more attention in the field of TDDSs because they can address the drawbacks of existing lipid-based systems, including protecting unstable APIs from denaturation and degradation and enabling a continuous drug release to minimize side effects. An increase in the gradient of concentration enhances the drug’s transdermal penetration. Because these NPs might be difficult to degrade, APIs can be kept for a very long time before being released from the NPs and diffusing into the skin’s deeper layers.

#### 7.2.3. Vesicles [95]

Vesicles are water-filled colloidal particles made up of molecules that are amphiphilic in a bilayer configuration. These amphiphilic molecules produce concentric bilayers with one or more shells (multilayer vesicles) when there is an excess of water present. Drugs that are soluble in fat or water can be delivered in vesicles for transdermal absorption. Vesicles can be employed topically to deliver a continuous release of stored drugs. The vesicles in TDDSs can also be used to regulate the absorption rate via a multilayer structure. Due to the presence of many components, vesicle systems can be classified according to the characteristics of the constituent substances into several categories, such as liposomes, transfersomes, and ethosomes. The advantages of various delivery methods are shown in Table 5.

## 8. The Evaluation of a TDDS

The development of a TDDS necessitates a thorough and systematic evaluation at several phases. Various evaluation processes are shown in Table 6.

### 8.1. Physicochemical Evaluation—Adhesive Evaluation

Three types of TDDS adhesion tests are generally mentioned in the United States Pharmacopoeia (USP). They include peel adhesion tests, release liner peel tests, and tack tests (the probe tack test and rolling ball method). The pressure-sensitive adhesives are evaluated for the following properties.

#### 8.1.1. Peel Adhesion Properties [104]

Peel adhesion refers to the amount of force needed to detach an adhesive covering from a designated surface for testing purposes. An essential characteristic of TDDSs is that the adhesive must ensure sufficient adherence of the device to the skin without causing any harm when it is removed. The molecular weight of the adhesive polymer, the kind and quantity of additives, and the composition of the polymer all have an impact on these qualities. The testing involves quantifying the amount of force needed to extract a solitary tape with a coating, which is affixed to a substrate, at a 180° inclination. The absence of any residue on the substrate signifies “adhesive failure”, which is a desirable outcome for TDDSs. The presence of remnants on the substrate suggests a “cohesive failure”, which indicates a lack of cohesive strength in the coating.

#### 8.1.2. Tack Properties [105,106]

Tack refers to the capacity of a polymer to stick to a surface with minimal applied pressure. Finger pressure is crucial in TDDSs. The adhesive strength of the tack is influenced by the molecular weight and content of the polymer, as well as the incorporation of tackifying resins into the polymer. Tests conducted to evaluate the performance of tack include the following:

(a) Thumbtack Test [105,106]

This is a subjective test in which the evaluation is conducted by pressing the thumb briefly into the adhesive.

(b) Rolling Ball Tack Test [105,106]

This experiment measures the distance of a stainless steel ball as it moves along an adhesive surface facing upwards. The lesser the tackiness of the adhesive, the greater the distance the ball will travel.

(c) Quick Stick or Peel Tack Test [105,106]

The peel force necessary to separate the adhesive from the substrate is quantified by applying a 90° angle force to the tape, peeling it away from the substrate at a rate of 12 inches per minute. The force is measured and documented as the tack value, which is denoted in ounces or grams per inch width. Higher values correspond to more tackiness.

(d) Probe Tack Test [105,106]

The probe makes contact with the glue, resulting in the formation of a bond between them. Tack is the measurement of the force, expressed in grams, needed to separate a probe from an adhesive at a constant rate.

#### 8.1.3. Shear Strength Properties [107]

This quantifies the adhesive polymer’s ability to stick together. The sufficient adhesive strength of a device ensures that it remains in place during application and does not leave any residue upon removal. The molecular weight and the type and quantity of the tackifier used have an impact on it. Shear strength or creep resistance is assessed by measuring the duration required to detach an adhesive-coated tape from a stainless steel plate while applying a predetermined weight that pulls the tape in a direction parallel to the plate.

### 8.2. Patch Width [108]

The width of the patch is determined using a digital micrometer, while the average thickness ensures the desired thickness of the created patch. The film thickness is measured using a screw gauge and a microscope dial gauge at various locations on the film.

### 8.3. Folding Endurance [109]

A section of the TDDS is cut and subsequently folded multiple times in a manner like a plug until it eventually fractures. The durability of the patch is determined by the number of times it can be folded from the same spot until it breaks.

### 8.4. Percentage of Moisture Content [110]

Every individual transdermal patch is individually weighed and then kept at room temperature for a duration of 24 h in desiccators with fused calcium chloride. After 24 h, the patches are weighed again, and the moisture content percentage is determined using the following formula:

$$\%\;moisture\;content=\frac{Initial\;weight-Final\;weight}{Initial\;weight}$$

### 8.5. Moisture Uptake [111]

The patches are stored in a desiccator at room temperature for a duration of 24 h. After a duration of 24 h, the patches are taken off and exposed to a concentrated solution of potassium chloride in desiccators at a relative humidity of 84% until they attain a stable weight. The calculation of the moisture uptake percentage is determined by the following:

$$\%\;moisture\;uptake=\frac{Final\;weight-Initial\;weight}{Initial\;weight}$$

### 8.6. Content Uniformity Test [112]

A total of 10 patches were selected for the purpose of this study, and the specific content of each patch was determined. The content uniformity test is considered acceptable if nine out of ten patches have a content uniformity ranging from 85% to 115%, and if the remaining individual patch falls within the limit of 75% to 125%. An additional examination is conducted on 20 patches, with their acceptable range set at 85% to 115%. If the results fall within this range, the test is considered successful.

### 8.7. Drug Content [113]

The patch is dissolved in a solvent following its cutting to the desired dimensions. Subsequently, the solution is passed through a filter media, and the constituents are identified using an appropriate analytical technique, such as a UV–visible spectrophotometer or high-performance liquid chromatography.

### 8.8. In Vitro Drug Release [114]

In vitro studies assist in the design and development of TDDSs. These investigations can aid in investigating how the drug is absorbed via the skin before its formulation into a transdermal therapeutic system. The data acquired from in vitro investigations of the developed TDDS can be utilized to optimize the formulation prior to conducting more expensive in vivo tests.

For this study, excised skin is placed on skin permeation cells. The use of excised skin is commonly preferred for in vitro investigations due to the fact that the SC, which is a biologically inactive tissue, serves as the primary barrier for drug permeation. The diffusion through the SC occurs through a passive process. The use of hairless mouse skin is advantageous due to the absence of any potentially harmful hair removal procedures. Additionally, this species is readily accessible and allows for the excision of skin specimens immediately prior to the permeation study. An appropriately designed in vitro apparatus ensures that the drug delivery mechanism originates exclusively from the TDDS. The paddle over disc method (USP apparatus V) is used for the in vitro assessment of the TDDS. Examples of in vitro membrane permeation apparatuses are the Valia–Chien (V-C) cell, Ghannam–Chien (G-C) membrane permeation cell, Jhawer–Lord (J-L) rotating disc system, Franz diffusion cell, and the Keshary–Chien (K-C) cell. The Franz diffusion cell and the K-C cell are the most commonly utilized among them. The K-C cell, a modified iteration of the Franz diffusion cell, possesses a receptor volume of 12 mL and a skin surface area of 3.14 cm^2^. The receptor solution is agitated by a magnet that rotates at a consistent pace of 600 revolutions per minute, powered by a 3-watt synchronous motor. The heater, which is regulated by a thermostat, maintains a temperature of 32 ± 0.5 °C. Periodically, a specific amount of the sample is taken from the receptor compartment and analyzed with a spectrophotometer.

### 8.9. Skin Irritation Study [115]

Skin irritation and sensitization studies can be carried out on healthy rabbits. The hair of the animal is removed from the dorsal side. The surface is cleaned with a rectified spirit and the patch is placed on the skin. After 24 h, the patch is removed. Depending on the degree of skin injury, the skin condition is assessed.

### 8.10. Stability Study [116]

These studies are conducted in compliance with ICH guidelines for 6 months. The samples are kept at 40 ± 5 °C and 75 ± 5% relative humidity and are analyzed at various time intervals, such as 0, 30, 60, 120, and 180 days.

### 8.11. In Vivo Evaluation

Studies on in vivo skin penetration may be conducted for the following objectives:

- To confirm and measure a transdermal drug’s systemic bioavailability.
- To confirm and measure a drug’s cutaneous bioavailability when applied topically.
- To determine whether several topical formulations of the same API are bioequivalent.
- To ascertain the frequency and severity of the systemic toxicological risk that may arise from the topical administration of a specific drug or formulation.
- To connect the drug’s resulting blood levels in humans to the treatment’s overall systemic effects [117].

In vivo evaluations of TDDSs can be carried out using animal models, human volunteers, and biophysical models.

#### 8.11.1. Animal Models [118,119,120,121]

Animal models are favored due to the substantial time and resources needed to conduct investigations in humans. The species utilized for both in vivo and in vitro testing encompass mouse, rat, guinea pig, rabbit, hairless mouse, hairless rat, cat, dog, tiny pig, horse, goat, squirrel, and rhesus monkey. Multiple experiments have been conducted to ascertain which animal models offer the most precise predictions of the device’s performance when tested on humans. The conducted experiments have resulted in the subsequent findings:

Small, hirsute animals are not highly effective prediction models for human in vivo transdermal drug administration. The penetration values seen in these animals exceed those observed in humans. This observation is corroborated by in vitro measurements. Shaving or depilating the skin of these animals for investigation can cause alterations in the skin’s resilience. The rhesus monkey is the most reliable model for the in vivo assessment of TDDSs. The application site is typically the forearm or abdomen, as these areas tend to have the least amount of hair on the animal’s body. The drawbacks associated with utilizing this particular animal species encompass the cost, necessary handling skills, and challenges related to accessibility. The utilization of rhesus monkeys is further restricted due to ethical reasons. Promising alternative animal models for predicting transdermal API distribution in humans include weanling pigs and nude mice with human skin grafts. The challenge faced while utilizing these animals lies in their unavailability and the necessary facilities and expertise needed for their handling.

#### 8.11.2. Human Models [122,123,124,125]

The last phase in the advancement of a transdermal device entails gathering pharmacokinetic and pharmacodynamics data subsequent to the administration of the device on human participants. A human-based in vivo assessment should provide the relevant data with minimal risk to the participants within a suitable timeframe. This method entails assessing percutaneous absorption by the indirect approach of quantifying radioactivity in waste products from the body after applying the labelled drug topically. To determine the absorbed drug percentage, it is necessary to assess the elimination that occurs after the API is administered parenterally. Optimal patient compliance is essential for the successful completion of the process, since it typically spans a duration of 5–7 days. This approach can be extremely beneficial for researching penetrants, including steroids and cosmetics. The percentage of the transdermal absorption rate is subsequently determined as follows:

$$\%\;dose\;absorbed=\frac{Total\;radioactivity\;excreted\;after\;topical\;administration}{Total\;radioactivity\;excreted\;after\;intravenous\;administration}\times 100$$

### 8.12. Cutaneous Toxicological Evaluation [126]

This can be performed by evaluating contact dermatitis. Contact dermatitis can be either contact irritant dermatitis or contact allergic dermatitis.

Contact irritant dermatitis results from direct toxic injury to the cell membrane, cytoplasm, or nuclei. Two types of protocols are used for evaluation: a ten-day primary irritation test and a twenty-one-day irritation test.

Contact allergic dermatitis involves a host immunological reaction to an antigen. It can be screened by the guinea pig maximization test.

## 9. Potential Applications of TDDSs

With the progress of technology and research, a wide range of prospective uses for transdermal therapeutic systems have been investigated. The TDDS significantly influences the administration of different APIs in the areas of pain control, central nervous system ailments, cardiovascular illnesses, and hormone treatments [127]. The United States approved the first transdermal patch containing scopolamine for systemic delivery in 1979. This patch was specifically designed to alleviate motion sickness. The Transderm-Scop four-layer system is a round, flat patch with a thickness of 0.2 mm and an area of 2.5 cm^2^. The product includes 1.5 mg of scopolamine and is specifically formulated to administer roughly 1 mg of scopolamine consistently through the microporous membrane that controls the release rate [128]. Additionally, there are other over-the-counter (OTC) TDDS products, including nicotine, capsaicin, and menthol. The Japanese market approved the first antihistamine transdermal patch, emedastine difumarate, in 2018 for the treatment of allergic rhinitis. Its impact is enduring, persisting for a duration of 24 h following administration. The FDA has recently granted approval for the use of ruxolitinib cream, the first topical Janus kinase inhibitor (JAK), in the treatment of mild-to-moderate atopic dermatitis. Hyaluronic-based systems are gaining popularity due to their widespread use in the pharmaceutical industry, where they are valued for their improved permeability and biocompatibility. Nicotine TDDSs deliver a continuous supply of nicotine into the bloodstream, serving as a replacement therapy to aid patients in achieving and maintaining cessation from smoking. The patches available commercially contain nicotine doses ranging from 7 to 21 mg. These patches are meant to be used daily for a period of treatment lasting between 6 and 12 weeks [129].

TDDSs for gene therapy: Using TDDSs, genetic material can be delivered to damaged cells in a manner similar to gene therapy. The near-infrared dye IR820 and p53 DNA-loaded TDDSs were produced, and the matrix was built using hyaluronic acid. The device quickly and effectively penetrated the SC, dissolved rapidly and released p53 DNA and IR820 at the locations of subcutaneous tumors. Because gene therapy and photothermal agents work well together, the patch demonstrated a strong anti-tumor impact in vivo [130].

TDDSs for cardiovascular disorders: To increase its bioavailability, propranolol, a non-selective beta-adrenergic blocker, is prepared as a TDDS. It attains around 23% oral bioavailability after first pass metabolism. After an initial lag time of 8 h, the propranolol TDDS reaches a steady-state plasma concentration of 9.3 ng/mL, indicating a bioavailability of around 75% compared to oral propranolol. For the treatment of aortic dissection, atrial fibrillation, premature ventricular contraction, and orthostatic hypotension brought on by heart failure, another TDDS containing bisoprolol is utilized (Bisono Tape). The initial transdermal system for hypertension, Catapres TTS, an antihypertensive drug (the clonidine transdermal therapeutic system), is offered in several sizes, wherein the quantity of the API discharged is directly related to the dimensions of the patch [131]. The clonidine stored in the reservoir starts to pass through the rate-controlling membrane and the skin, entering the systemic circulation. Additionally, it is used to treat drug withdrawal syndrome and attention deficit hyperactivity disorder. An angiotensin II receptor blocker, losartan, is also being developed as a TDDS, and its bioavailability is increased 1.93 fold over that of oral losartan. Another medication that is important to discuss in cardiovascular therapy is nitroglycerin. To treat angina pectoris, a nitroglycerin TDDS was created. The substance has a short plasma half-life and reaches elevated levels in the bloodstream. When ingested orally, it undergoes fast hepatic metabolism and the transdermal approach circumvents this first pass effect. A number of nitroglycerin-containing TDDSs have been developed, including Minitran, Nitro-Dur, Nitro-Dur II, Transder-Nitro, and Nitrodisc. These products maintain nitroglycerin drug delivery for 24 h after application [132].

TDDSs for vaccination: A microneedle-based smallpox vaccine was created by researchers, providing a more convenient and painless injection substitute. Furthermore, they created an influenza vaccination targeting skin antigen-presenting cells using a lytic microneedle-based TDDS. The microneedles were created using a biocompatible polymer that contained an inactivated influenza virus vaccine, which could be inserted and dissolved into the skin within a few minutes [133].

TDDSs for infectious diseases: To formulate the transdermal antibodies, the zwitterionic characteristic of cephalexin was incorporated in solid lipid nanoparticles to develop a TDDS of cephalexin [134]. With minimal antibiotic usage, this formulation demonstrated a consistent antibacterial action. For the TDDS, tetracyclines were added to hydrogel-forming microarray patches. Amoxicillin, ampicillin, and kanamycin were loaded into bacterial cellulose/polycaprolactone patches for the development of the transdermal administration. These techniques have a strong bactericidal effect on *Escherichia coli* and *Staphylococcus aureus* [135].

TDDSs for insulin delivery: The development of TDDS for insulin delivery presents a number of obstacles. The pancreas secretes the hormone insulin, which is essential for controlling the blood sugar levels. In order to treat diabetes, transdermal insulin delivery patches are utilized to transfer insulin through the skin and into the bloodstream. In place of more conventional insulin administration techniques like injections and insulin pumps, transdermal insulin delivery can offer a practical and affordable option. These devices deliver a steady dose of insulin over a specific period of time through the skin on the thigh, abdomen, or upper arm. Large protein molecules like insulin are difficult for the skin to absorb. In order to get around this, a novel method of transdermal protein administration has been created, which uses a water-swellable spherical double-layered microneedle patch at the tip. Because of their unique design, microneedles can selectively enlarge distally after being inserted into the skin, enabling them to mechanically engage the soft tissue. The TDDS of insulin has the potential to provide an effective and convenient method to deliver insulin to diabetic patients [136].

TDDSs for central nervous system (CNS) disorders: These TDDSs have a favorable pharmacological profile and bioavailability and offer a prolonged therapeutic dosage at plasma levels. Targeting monoamine oxidase, the selegiline (Emsam) TDDS is a selective and irreversible monoamine oxidase inhibitor used to treat Parkinson’s disease. The Food and Drug Administration (FDA) approved the first Parkinson’s patch containing rotigotine in 2007. This patch is designed to be applied once a day. Methylphenidate is a transdermal device with an adhesive-based matrix. It is specifically used to treat attention deficit hyperactivity disorder (ADHD) in children [137]. It is offered in different dosages and administers the specified dose over a duration of 9 h. The drugs donepezil, galantamine, and rivastigmine, which are cholinesterase inhibitors and improve cholinergic transmission in mild-to-severe Alzheimer’s disease, were also developed as TDDSs [138].

TDDSs for hormonal deficiencies and contraception: The Ortho Evra transdermal system is a contraceptive patch of the thin matrix type that includes 6 mg of norelgestromin and 0.75 mg of ethinyl estradiol. It releases the specified amount into the bloodstream every 24 h [139]. The testosterone TDDSs known as Testoderm and Androderm are offered in different dosages for the purpose of hormone replacement therapy in males with testosterone insufficiency. The estradiol hormone, in the form of the Estraderm TDDS, is used for managing the moderate-to-severe vasomotor symptoms related to menopause, female hypogonadism, primary ovarian failure, and atrophic diseases. The TDDS provides a constant release of estradiol when applied to unbroken skin through the rate-limiting barrier [140]. Examples of various TDDSs are summarized in Table 7. Various new drugs approved as TDDSs are given in Table 8.

## 10. Quality by Design (QbD) Approach to TDDSs

The optimization of TDDSs is achieved through the use of experimental design techniques [151]. The approach focuses on investigating the impact of temperature and occlusion on drug flux by examining the stability of thermodynamic changes, crystallization, and polymer cross-linking. Utilizing the QbD methodology in the production of TDDSs assures the excellence, security, and effectiveness of the end pharmaceutical product, resulting in an effective formulation optimization [3]. Design of experiment (DoE) enhances the quality of the final formulation in this particular instance by minimizing the number of necessary trials. These experiments have been verified using various mathematical models. The models employed include the Box–Behnken design, central composite rotatable design, surface response composite design, and randomized fractional factorial design [152]. The ANOVA statistically confirmed the factors that had a substantial impact on the dependent variables. The implementation of QbD in the creation of pharmaceutical products leads to enhanced manufacturing and development efficiency, as well as the identification of potential dangers and characteristics that may impact product quality [153].

Recently, a number of researchers have been utilizing the QbD methodology in the creation of TDDSs in order to obtain superior pharmaceutical goods [154]. This is accomplished by comprehending and managing the formulation, materials, and manufacturing variables. The application of QbD has been utilized to enhance drug release and targeting, as well as to optimize the pharmacokinetics and pharmacodynamics features of TDDSs [155].

### 10.1. Quality Target Product Profile (QTPP)

The applicant should determine the desired QTPP before moving on with TDDS development. The QTPP is a potential description of the TDDS product’s quality attributes that, while accounting for product safety and efficacy, should ideally be attained to guarantee the intended quality. The QTPP components and their quality factors for TDDSs could consist of the in vivo delivery of active ingredients to achieve the desired therapeutic effect, residual drug minimization, adherence for the duration of the wear period, the minimization of irritation, physical and chemical stability, and non-drug substance-related impurities. Depending on the patient demographic, treatment need, or other functional property demands, there might be additional QTPP components. For instance, the finished product’s size may be a QTPP element depending on where the product is to be placed on the body if the patient population is pediatric [156].

### 10.2. Critical Quality Attributes (CQAs)

**Figure 5:**
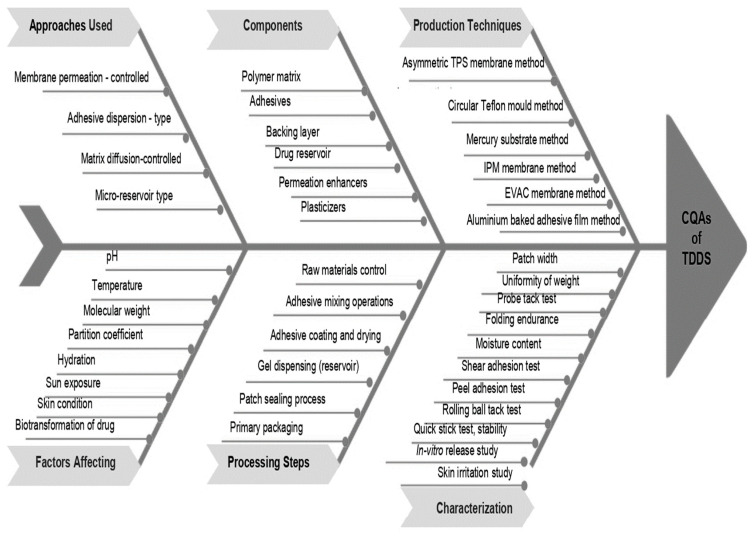
Ishikawa (fishbone) diagram to assess critical quality attributes (CQAs) of TDDSs.

CQAs are generally related to the selection of the correct amount of excipients and APIs, which affects the performance of the product [157]. The critical quality attributes are described in a fishbone or Ishikawa diagram, as shown in Figure 5.

A CQA is a physical, chemical, biological, or microbiological property or characteristic that should be within an appropriate limit, range, or distribution to ensure the desired product quality [158]. The list of product CQAs can be created using the product’s QTPP in conjunction with past information, risk assessments, and/or experiments. Every CQA should be related to one or more components of the TDDS product’s QTPP, either alone or in conjunction with one or more other CQAs. As new information is discovered and product development moves forward, the list of CQAs may be adjusted. The application should include the CQAs for the drug substance(s), components, excipients, and container closure system. The CQAs for TDDSs typically include the following: peel adhesion, tack, release liner peel strength, shear strength, cold flow, residual solvents, residual monomers, microbial limits, the uniformity of dosage units, assay, permeation enhancer content, impurities and degradants, in vitro drug release profile, and (if present) preservative/antioxidant content. The physicochemical and biological characteristics of a drug substance should be taken into consideration when choosing the drug, because they can affect the TDDS product’s performance and manufacturing feasibility. Molecular weight, melting point, partition coefficient, pKa, water solubility, pH, and other characteristics that affect the rate of distribution should be taken into account. A TDDS may contain a variety of adhesives, permeation enhancers, rate-controlling or non-rate-controlling membranes, solubilizers, plasticizers/softeners, or tackifiers, among other ingredients. These ingredients can all affect the TDDS’s performance and quality attributes [159].

## 11. Regulatory Guidance of TDDSs

The design of the systems might vary from drug-in-adhesive matrix systems to more intricate systems that necessitate microelectronics. Several guidelines were issued about the advancement of TDDS concerns. Transdermal products possess characteristics that can potentially cause skin irritation and/or sensitization. These effects may be attributed to either the delivery method itself or the system working together with the drug component. In order to reduce these adverse occurrences, the guidance presented recommendations that were necessary to carry out during the process of drug development [160,161].

Current TDDSs and topical patches contain an excessive quantity of the API compared to the desired amount to be administered to the patient. An additional quantity of the drug substance is required to ensure the efficient delivery of the desired drug dosage to the patient. This excess drug remains in the system after use. The presence of residual drug substances in TDDSs and topical patches has the potential to significantly affect the quality, effectiveness, and safety of these products, including the risk of abuse. Therefore, it is imperative to guarantee the utilization of a suitable scientific approach in the design and development of these items. The strategy should aim to decrease the remaining drug substance to the greatest extent possible while taking into account the current technological capabilities [39].

If the active ingredient may achieve the desired clinical impact through a different pharmacokinetic profile than that of an immediate release form, then a prolonged release dosage form may be deemed suitable. A sustained release formulation may provide several benefits compared to an immediate release form. This guidance offers suggestions for the planning and execution of research aimed at assessing the adhesive efficacy of a TDDS or a topical patch, which is being presented to support an Abbreviated New Drug Application (ANDA) [162].

Implementing a Quality by Design (QbD) strategy can streamline the process of developing TDDSs and topical patches that have been specially designed to meet the needs of patients and address post-use considerations. Specifically, it can assist in the development of a product that delivers the optimal dosage of an API through the skin while decreasing the drug load, hence reducing the remaining amount of the API. Quality by Design (QbD) applies to both the development of new products and the process of reformulating current products. Furthermore, it can result in the enhanced comprehension and continual improvement of the product during its entire lifespan [163].

A TDDS must adhere to numerous regulatory standards to guarantee its quality, safety, and efficacy. Below are the essential points from the guidelines [164]:

1. Quality requirements: The European Medicines Agency (EMA) offers comprehensive standards about the quality of transdermal patches. These encompass specifications for the description, development, production, and regulation of the pharmaceutical product.
2. Pharmaceutical development: This includes formulation development, stability program development, and both in vitro and in vivo performance testing of the drug product.
3. Manufacturing process: The guidelines encompass the formulation of the manufacturing process, which includes the regulation of excipients, laminates, and liners.
4. Bioequivalence: For generic TDDSs, bioequivalence must be established, incorporating pharmaceutical equivalence and analogous bioavailability.
5. Legal foundation and applications: The guidelines delineate the legal foundation for new applications, encompassing the description and content of the drug product, as well as the prerequisites for supporting generic or abridged applications.

These criteria guarantee that TDDSs are designed and produced to rigorous standards, ensuring their safety and efficacy for patients.

## 12. Clinical Considerations for the Use of a TDDS

The patient must be advised of the following general guidelines while using a TDDS [165,166,167,168,169,170].

- The extent of percutaneous absorption can differ depending on the location of the application. The intended primary application location is indicated in the package insert for each product. The patient should be informed about the significance of utilizing the prescribed location. Rotating sites reduces the chance of skin irritation and allows the skin beneath a patch to return to its usual permeability properties after being occluded. After a period of one week, it is possible to reuse skin sites.
- The use of a TDDS should be limited to skin that is clean, dry, and devoid of hair. Additionally, the skin should not be oily, irritable, inflamed, damaged, or callused. Increased skin moisture can enhance the rate of drug penetration beyond the targeted level. The presence of excess sebum on the skin can hinder the ability of the patch to stick to the intended area.
- The application of skin lotion at the location of the application should be avoided. Lotions alter the moisture of the skin and have the potential to modify the partition coefficient between the API and the skin.
- It is not advisable to cut TDDSs (to decrease the dose) as this compromises the integrity of the system.
- The unit should be removed from its protective packaging, taking caution to avoid any tearing or cutting. The protective backing should be carefully removed to reveal the sticky layer, making sure not to touch the adhesive surface with one’s fingertips. To achieve consistent contact and adherence, it is necessary to firmly apply pressure to the skin location using the heel of the hand for approximately 10 s.
- The TDDS should be positioned in a location where it is not susceptible to friction from clothing or bodily motion. It is permissible to keep it on while showering, bathing, or swimming. If a TDDS becomes dislodged before the intended time, one can either try to reapply it or replace it with a new system.
- It is important to wear a TDDS for the entire duration specified in the product’s instructions. Subsequent to that time frame, it ought to be eliminated and substituted with a new system as directed.
- The patient must be advised to properly cleanse their hands before and after applying a TDDS. Using precautions and avoiding touching the eyes or mouth while handling the system is important.
- If the patient experiences sensitivity or intolerance to a TDDS, or if skin irritation occurs, the patient should seek reevaluation.
- When removing a used TDDS, it should be folded in half with the adhesive layer to prevent any possibility of reuse. The patch that has been used, and still contains traces of the API, should be placed inside the pouch of the replacement patch and disposed of in a way that is safe for children and pets.
- Apart from the guidelines regarding the location, duration, and disposal of TDDS patches, clinicians also need to consider taking into account possible issues with cutting TDDSs to modify dosage, safety issues regarding the electrical conductivity of metal-containing patches, suitable approaches for handling patch adhesion failures, and the advisability of writing on patches for medication safety or compliance purposes. Additionally, clinicians need to be ready to advise patients with TDDS-specific guidelines regarding limiting sunlight and other heat sources while wearing a TDDS.

## 13. Conclusions and Future Challenges

This article provides valuable insights into the TDDS, with evaluation techniques and therapeutic applications, serving as a convenient reference for research scientists engaged in transdermal drug delivery system development.

The TDDS is a highly valuable means of administering APIs, offering numerous advantages compared to alternative delivery routes. It has the ability to circumvent the digestive system and initial metabolism in order to achieve sustained drug release over a prolonged duration, resulting in enhanced therapeutic effectiveness. The TDDS is a method of delivering APIs that does not require intrusive procedures. It can be used on age groups who are more susceptible to harm, such as children and elderly patients. The large surface area and easy access to the skin make it a handy and patient-friendly target for drug delivery. The physical methods employed in the administration of transdermal therapeutic systems are designed to deliver a diverse array of APIs, particularly those that are water-soluble and large in size. However, these drug delivery systems encompass the potential for toxicity resulting from incorrect dosage, inadequate adherence, limited drug permeation, skin irritation, or patch malfunction. It is limited to lipophilic, low-molecular weight APIs due to the barrier properties of human skin.

The transdermal method of drug administration is gaining widespread acceptance due to recent technological advancements and the ability to deliver APIs directly to the site of action without breaking the skin membrane. Nevertheless, transdermal technologies are constrained by the relatively impenetrable thickness of the outer SC layer. Scientists are attempting to overcome this obstacle of low permeability with physical and chemical methods. These technologies have demonstrated considerable potential in recent years and are increasingly widespread in the field of healthcare. The intricate nature of chemical TDDSs hinders their commercialization. Furthermore, chemical techniques have a significant limitation in terms of transporting hydrophilic macromolecules, such as proteins. However, only a limited number of commercially available TDDSs have employed physical methods, particularly for hydrophilic drugs and macromolecules. They possess substantial potential as they enable the development of effective treatments using both hydrophobic and hydrophilic APIs.

There are several facets to TDDSs’ future development. Some of the aspects envisioned include the development of better skin permeability enhancers, the widespread usage of redesigned drug molecules (e.g., prodrugs), and improvements to currently available devices to give optimal blood levels. A better understanding of the composition of the SC barrier, the way enhancers interact with it, and the creation of structure–activity connections for enhancers will help achieve the best possible results with the fewest amount of toxic effects. Penetration enhancers will be crucial in the future development of more efficient and reasonably priced TDDSs. The primary obstacle is incorporating macromolecules, such as proteins, short interfering RNA, and other biotechnology products, into transdermal delivery systems. Approaches including electroporation, iontophoresis, sonophoresis, and microneedles are also being used to address this difficulty. This type of transdermal delivery has several benefits, such as delivering less potent medications, using smaller patches to lessen the issue of local irritation, managing the dosage by adjusting the applied electric current density, and patient-specific systems. User acceptance will probably depend on success in miniaturizing the assembly. Another strategy is to use nanotechnology to create sophisticated therapeutic tools. This method is gaining more scientific attention because it has several benefits over topical dermatotherapy.

Enhancing our understanding of diverse biological interactions and polymer mechanisms is necessary to optimize this drug delivery system. It has garnered significant attention due to its numerous advantages compared to traditional drug delivery methods. Further research and development are necessary to enhance the safety and effectiveness of this drug delivery technology.

## Figures and Tables

**Table 1:** Advantages and limitations of a TDDS [11,12,13,14].

Advantages	Limitations
The concentration and dose frequency of the drug can be reduced due to improved bioavailability, protecting sensitive drugs from the harsh conditions of the GIT; circumvents first pass effect of drugs	The drug must have some desirable physicochemical properties (including its molecular weight, solubility, partition coefficient, and dissociation constant) for penetration through the SC
A simplified dosage regimen leads to improved patient compliance and reduced inter- and intra-patient variability	Skin irritation or contact dermatitis at the site of application due to the drug or the excipients and enhancers of the drug used to increase percutaneous absorption
Can be used for chronic conditions that require drug therapy for a long period; maintains a steady-state plasma drug concentration over an extended time	The barrier function of the skin changes from one site to another on the same person, person to person and with age. Variability of application site conditions
Prevents the hassle of parenteral therapy since TDDSs are non-invasive	Only potent and low-dose drugs are suitable for transdermal drug delivery
Failure to produce the therapeutic effect associated with intermittent dosing can also be avoided	Unsuitable for large molecule (M.Wt above 500 Daltons) drugs that metabolize in the skin and undergo protein binding in the skin
Reduces systemic drug interactions and self-administration is possibleThe drug can be terminated at any point of time by removing the transdermal patch	The therapeutic efficacy of the dosage form can be affected by cutaneous metabolism The dosing option is limited

**Table 2:** Polymers used in TDDSs [50].

Natural Polymers	Synthetic Elastomers	Synthetic Polymers
Cellulose derivatives, zein, gelatin, waxes, shellac, gums and their derivatives, proteins, natural rubber, and starch	Polybutadiene, hydrin rubber, polysiloxane, silicone rubber, butyl rubber, acetonitrile, styrene-butadiene rubber, neoprene, and nitrile	Polyvinyl chloride, polyvinyl alcohol, polyacrylate, polypropylene, polyethylene, polyamide, polyuria, polymethyl methacrylate, and epoxy and polyvinyl pyrrolidone

**Table 3:** General classification of permeation enhancers [55].

Solvents	Surfactants	Binary Systems	Miscellaneous Compounds
They increase penetration by swelling the polar pathway and by fluidizing lipids. Examples: Water, alcohols, alkyl methyl sulfoxides, dimethyl acetamide, dimethyl formamide, pyrrolidones, propylene glycol, glycerol, silicone fluids, and isopropyl palmitate.	They are used to enhance the polar pathway transport, especially of hydrophilic drugs. These compounds are, however, skin irritants. Anionic surfactants can penetrate and interact strongly with the skin and can induce large alterations in the skin. Cationic surfactants are more irritant than anionic surfactants; hence, they have not been widely used as skin permeation enhancers. Of the three classes of surfactants, nonionic surfactants have been recognized as those with the least potential for irritation and are widely used. Examples: Anionic surfactants: dioctyl sulphosuccinate, sodium lauryl sulphate, and decodecylmethyl sulphoxide. Nonionic surfactants: Pluronic F127 and Pluronic F68. Bile salts: sodium taurocholate, sodium deoxycholate, and sodium tauroglycocholate.	These systems open up the heterogeneous multilaminate pathway, as well as the continuous pathways. Examples: Propylene glycol-oleic acid and 1, 4 butane diol-linoleic acid.	These include urea (a hydrating and keratolytic agent), N, N dimethyl-m-toluamide, calcium thioglycolate, eucalyptol, and soyabean casein.

**Table 4:** Examples of membrane permeation-controlled systems [63,64,65,66].

TDDS	Use
Nitroglycerin-releasing transdermal patch (Transderm-Nitro)	Once a day medication in anginal pectoris
Clonidine-releasing transdermal patch (Catapres)	7 days of therapy for hypertension
Estradiol-releasing transdermal patch (Estraderm)	Treatment of menopausal syndrome for 3–4 days
Scopolamine-releasing transdermal patch (Transderm-Scop)	72 h of prophylaxis for motion sickness

**Table 5:** Advantages of various delivery methods of TDDSs [96,97,98,99,100,101].

	Methods	Advantages
**Active delivery**	Iontophoresis	Improves the delivery of polar molecules and high-molecular weight APIs, easy to administer, and continuous or pulsatile delivery of APIs
	Sonophoresis	Strict control of transdermal diffusion rates, greater patient approval, less risk of systemic absorption, and non-sensitizing
	Electroporation	Highly effective, reproducible, rapid termination of drug delivery, and non-sensitizing
	Photomechanical waves	Improve the transfer of molecules across the plasma membrane without loss of viability and do not cause pain or discomfort
	Microneedles	Painless administration of the API, faster healing at the injection site, no fear of needle, and specific delivery of the APIs
	Thermal ablation	Avoids pain, bleeding, and infection; better control and reproducibility; low cost; and disposable devices
**Passive delivery**	Nanoemulsion	Long-term thermodynamic stability, excellent wettability, high solubilization capacity, and physical stability
	Polymeric nanoparticles	Targeted and controlled release behavior, high mechanical strength, and both hydrophilic and lipophilic APIs can be loaded
	Vesicles	Sustained drug release behavior and control the absorption rate through a multilayered structure

**Table 6:** Evaluation methods of TDDS [102,103].

Physicochemical Evaluation	In Vitro Evaluation	In Vivo Evaluation
Compatibility study	In vitro release study	Animal models
Thickness test	Skin irritation study	Human volunteers
Uniformity of weight	Stability study	Toxicological evaluation
Drug content		
Moisture content		
Adhesive evaluation		
Tensile strength		
Folding endurance		
Water vapor transmission study		
Microscopic study		

**Table 7:** Summary of TDDSs [7,8,32,141,142,143,144].

Drugs	Indications
Nicotine	Cessation of tobacco smoking
Fentanyl CII (Duragesic)	Moderate/severe pain
Buprenorphine CIII (Bu Trans)	Relief for severe pain
Oestrogen, Levonorgestrel, Estradiol	Treat menopausal syndromes, postmenopausal osteoporosis
Ortho Evra or Evra (norelgestromin, ethinyl estradiol)	Contraceptive
Nitroglycerin	Angina pectoris and relieves pain after surgery
Scopolamine	Motion sickness
Clonidine	Antihypertensive
MAOI selegiline	Antidepressant
Methylphenidate	Attention deficit hyperactivity disorder (ADHD)
Asenapine	Antipsychotic agent
Vitamin B12 (Cyanocobalamin)	Supplement
Rivastigmine, Donepezil	Alzheimer’s disease
Asenapine	Bipolar disorder
Bisoprolol	Atrial fibrillation
Clonidine	Hypertension, Tourette syndrome, ADHD
Dextroamphetamine	ADHD
Granisetron	Anti-emetic
Lidocaine	Treatment of pain
Oxybutynin	Overactive bladder
Rotigotine	Parkinson’s disease
Testosterone	Hypogonadism in males
Selegiline	Depression

**Table 8:** New drug approval for TDDSs [80,145,146,147,148,149,150].

Drug Name	Formulations	Approval Year	Use
Tazarotene	Lotion	2019	Acne
Asenapine	Transdermal system	2019	Schizophrenia
Trifarotene	Cream	2019	Acne
Tribanibulin	Ointment	2020	Actinic keratosis
Clascoterone	Cream	2020	Acne
Abametapira	Topical lotion	2020	Head lice removal
Calcipotriene and betamethasone dipropionate	Cream	2020	Plaque, psoriasis
Minocycline	Topical foam	2020	Rosacea
Lactic acid, citric acid, and potassium bitartrate	Vaginal gel	2020	Contraceptive
Ethinylesyradiol and levonoegesterol	Transdermal system	2020	Contraceptive
Ruxolitinib	Cream	2021	Atopic dermatitis
Butenafine hydrochloride	Cream	2021	Fungal skin infection
Fentanyl	Patch	2021	Pain
Tretinoin benzoyl peroxide	Cream	2021	Acne

## Data Availability

Not applicable.
